# Neurally adjusted ventilatory assist versus pressure support ventilation: a randomized controlled feasibility trial performed in patients at risk of prolonged mechanical ventilation

**DOI:** 10.1186/s13054-020-02923-5

**Published:** 2020-05-14

**Authors:** Daniel J. Hadfield, Louise Rose, Fiona Reid, Victoria Cornelius, Nicholas Hart, Clare Finney, Bethany Penhaligon, Jasmine Molai, Clair Harris, Sian Saha, Harriet Noble, Emma Clarey, Leah Thompson, John Smith, Lucy Johnson, Phillip A. Hopkins, Gerrard F. Rafferty

**Affiliations:** 1 Critical Care, King’s College Hospital, London, UK; 2 Centre for Human and Applied Physiological Sciences, King’s College London, London, UK; 3 Florence Nightingale Faculty of Nursing, Midwifery & Palliative Care, King’s College London, London, UK; 4 Sunnybrook Health Sciences Centre and Sunnybrook Research Institute, Toronto, Canada; 5 School of Population Health and Environmental Sciences, King’s College London, London, UK; 6 Faculty of Medicine, School of Public Health, Imperial College, London, UK; 7 Lane Fox Unit, Guy’s and St Thomas’ NHS Foundation Trust, London, UK

**Keywords:** Critical care, Weaning, Interactive ventilatory support, NAVA studies, Randomized controlled trial

## Abstract

**Background:**

The clinical effectiveness of neurally adjusted ventilatory assist (NAVA) has yet to be demonstrated, and preliminary studies are required. The study aim was to assess the feasibility of a randomized controlled trial (RCT) of NAVA versus pressure support ventilation (PSV) in critically ill adults at risk of prolonged mechanical ventilation (MV).

**Methods:**

An open-label, parallel, feasibility RCT (*n* = 78) in four ICUs of one university-affiliated hospital. The primary outcome was mode adherence (percentage of time adherent to assigned mode), and protocol compliance (binary—≥ 65% mode adherence). Secondary exploratory outcomes included ventilator-free days (VFDs), sedation, and mortality.

**Results:**

In the 72 participants who commenced weaning, median (95% CI) mode adherence was 83.1% (64.0–97.1%) and 100% (100–100%), and protocol compliance was 66.7% (50.3–80.0%) and 100% (89.0–100.0%) in the NAVA and PSV groups respectively. Secondary outcomes indicated more VFDs to D28 (median difference 3.0 days, 95% CI 0.0–11.0; *p* = 0.04) and fewer in-hospital deaths (relative risk 0.5, 95% CI 0.2–0.9; *p* = 0.032) for NAVA. Although overall sedation was similar, Richmond Agitation and Sedation Scale (RASS) scores were closer to zero in NAVA compared to PSV (*p* = 0.020). No significant differences were observed in duration of MV, ICU or hospital stay, or ICU, D28, and D90 mortality.

**Conclusions:**

This feasibility trial demonstrated good adherence to assigned ventilation mode and the ability to meet a priori protocol compliance criteria. Exploratory outcomes suggest some clinical benefit for NAVA compared to PSV. Clinical effectiveness trials of NAVA are potentially feasible and warranted.

**Trial registration:**

ClinicalTrials.gov, NCT01826890. Registered 9 April 2013.

## Background

Neurally adjusted ventilatory assist (NAVA) uses electromyographic signals from the diaphragm (Edi) detected via a specialized naso-gastric feeding catheter (Getinge, Solna, Sweden), as a measure of neural respiratory drive and as a means of controlling the delivery of inspiratory support by a mechanical ventilator [1]. Over the last 12 years, studies have suggested several important physiological benefits associated with NAVA, including improved breath synchronization and effective assist that is proportional to neural respiratory drive [2, 3]. Studies have not yet, however, demonstrated improved patient outcomes as was originally suggested [1], with only two, relatively small trials finding non-statistically significant effects on secondary outcomes [3, 4].

Although there is a demand for adequately powered trials of NAVA [5], uncertainties remain in relation to its use over prolonged durations. Estimates of intervention adherence and protocol compliance provide critical information for the calculation of statistical power [6]. Randomized controlled clinical trials are expensive [7], and poor adherence risks a type II error, with an intervention deemed ineffective when it was actually not delivered with sufficient fidelity [8, 9]. Although good NAVA mode adherence was observed up to 48 h from randomization in a recent efficacy RCT [3], conflicting results were reported in a subsequent physiological RCT where NAVA failed in 7/20 (35%) participants across the same time period. To date, no trial has investigated adherence to a NAVA trial protocol beyond 48 h or determined the reasons for adherence or lack thereof.

The primary aim of this study was, therefore, to assess the feasibility of a trial protocol comparing NAVA to PSV, via an assessment of mode adherence. Our primary feasibility objectives were to assess mode adherence (proportion of time adherent to assigned mode) and protocol compliance (binary—≥ 65% mode adherence) during the entire study period. In contrast to previous trials, recruitment was commenced soon after intubation (ability to trigger the ventilator was not a requirement for inclusion), and the intervention was continued to the end of MV support or D28. In addition, reasons for mode cross-over and poor adherence were recorded. Some study results have been previously reported in the form of abstracts [10, 11].

## Methods

An open-label, parallel-group, randomized feasibility trial was undertaken in four ICUs (surgical, general medical, neuro/trauma, and liver) comprising 75 beds at a university-affiliated hospital in London, UK. The trial was prospectively registered (NCT01826890) and was approved by the London Westminster ethics committee (13/LO/0012).

### Participants

The inclusion criteria were as follows: adults receiving invasive MV with at least one of the following risk factors for prolonged MV [12]: (1) COPD, (2) heart failure (HF), or (3) acute respiratory distress syndrome (ARDS). Diagnoses by senior grade specialist physicians (respiratory, cardiac, intensivist) or by a non-specialist physician combined with either objective test results (spirometry, a CT scan, lung biopsy, cardiac echocardiogram) and/or prescribed treatment were required prior to enrolment (Additional file 1). The exclusion criteria were as follows: expected extubation, death, or treatment withdrawal within 48 h; > 96 h from intubation; > 24 h of spontaneously triggered ventilation mode support; suspected or proven hypoxic brain injury; high spinal injury; severe traumatic brain injury; neurological cause of ventilator dependency such as Guillain-Barré syndrome or Myasthenia Gravis; contraindication to nasogastric tube insertion; requirement for domiciliary ventilation; enrolment in any other interventional clinical trial; non-English speakers with inadequate translation available to allow informed consent; and pregnancy.

Research staff obtained informed consent from the participant’s legally authorized representative or proxy, and participant consent was sought once capacity was regained as required by the UK law [13].

### Randomization

Using a 1:1 allocation ratio, treatment order was randomized with permuted blocks of random sizes using online randomization software, managed by an independent clinical trial unit [14]. The study intervention could not be blinded; treating clinicians, researchers, participants, families, and outcome assessors were aware of study group allocations.

### Procedures

In the NAVA group, NAVA catheters were inserted within 4 h of randomization. Clinicians were instructed to record the maximum Edi hourly, to use the NAVA mode in place of PSV, and to target an Edi of ≥ 8 μV by adjustment of sedation dose and/or adjustment of MV settings where appropriate. A lower target level of 8 μV was set following basic analysis of existing literature and local audit data to avoid over-ventilation and over-sedation. NAVA parameters were set by clinicians according to a pragmatic protocol (Additional file 1), which involved matching hypothetical to actual pressure delivery (NAVA preview mode) and a brief period of observation to ensure stability. In the PSV group, ventilation settings were adjusted according to tidal volumes, clinicians were advised to review cycling criteria to optimize synchrony, and participants were otherwise ventilated according to local practice.

In both groups, protective tidal volumes of between 6 and 8 ml/kg of predicted body weight were recommended (Additional file 2, Table 4). Weaning guidelines included performing an assessment of readiness for a spontaneous mode, gradual stepwise reduction in ventilation support, sedation limitation according to Richmond Agitation and Sedation Scale (RASS) targets [16], and daily consideration of sedation holds and spontaneous breathing trials determined by the clinical team. Extubation readiness and practice were determined by clinicians. A full description of the weaning process is provided in Additional file 1. Study documents also guided troubleshooting of NAVA technical difficulties. The protocol was continued for up to 28 days from randomization.

### Outcomes

The primary feasibility outcome was the proportion of MV time spent in assigned mode from randomization to extubation, death, or D28. Continuous MV (CMV) modes and CPAP were permitted in both groups, but no other exclusively spontaneously triggered modes were allowed. A priori, we considered a participant to be protocol compliant if mode adherence was ≥ 65% (binary) and the trial to be feasible if ≥ 65% of participants were compliant. Secondary feasibility outcomes were the proportion of participants with mode cross-over, duration of cross-over, reasons for cross-over, protocol acceptability (participant consent/physician refusal rates), and recruitment rates. Secondary exploratory outcomes included ventilator-free days (VFDs) to D28 and D90 and duration of MV from randomization; ICU, hospital, D28, and D90 mortality; ICU and hospital length-of-stay from randomization; mean RASS; and sedation dose and bolus dose frequency per infusion day. Safety outcomes included ventilator-associated pneumonia (VAP), pneumothoraxes, and incidence of unplanned extubation (Additional file 1).

### Sample size and statistical analyses

Using 65% as the lower bound of the confidence interval (CI) (the minimum acceptable proportion to indicate trial feasibility) and an expected attrition of 5% (i.e. no NAVA or PSV weaning), a sample size of 76 patients (38 in each arm) would estimate a protocol compliance of 75% to within a 95% CI of ± 10% [17]. Descriptive statistics were used to characterize the sample. Categorical data were compared using chi-square or Fisher exact tests and continuous variables using Mann-Whitney or independent samples *t* tests as appropriate. Effect sizes are reported as median difference (MD) using the Hodges-Lehmann estimating method [15] for continuous variables, and as relative risk (RR) for binary data [18]. Effect estimates are reported with 95% CIs [18, 19]. Time to breathing without assistance and live ICU discharge are displayed using Kaplan-Meier curves with log-rank tests. A two-sided *P* value of < 0.05 was considered statistically significant. Analysis of clinical outcomes was exploratory and uncorrected for multiple comparisons; therefore, the statistically significant results could be due to chance. Qualitative descriptions of cross-over were categorized using content analysis [20]. Statistical analyses were performed using GraphPad Prism version 7.04 (GraphPad Software, La Jolla, CA, USA).

## Results

**Fig. 1:**
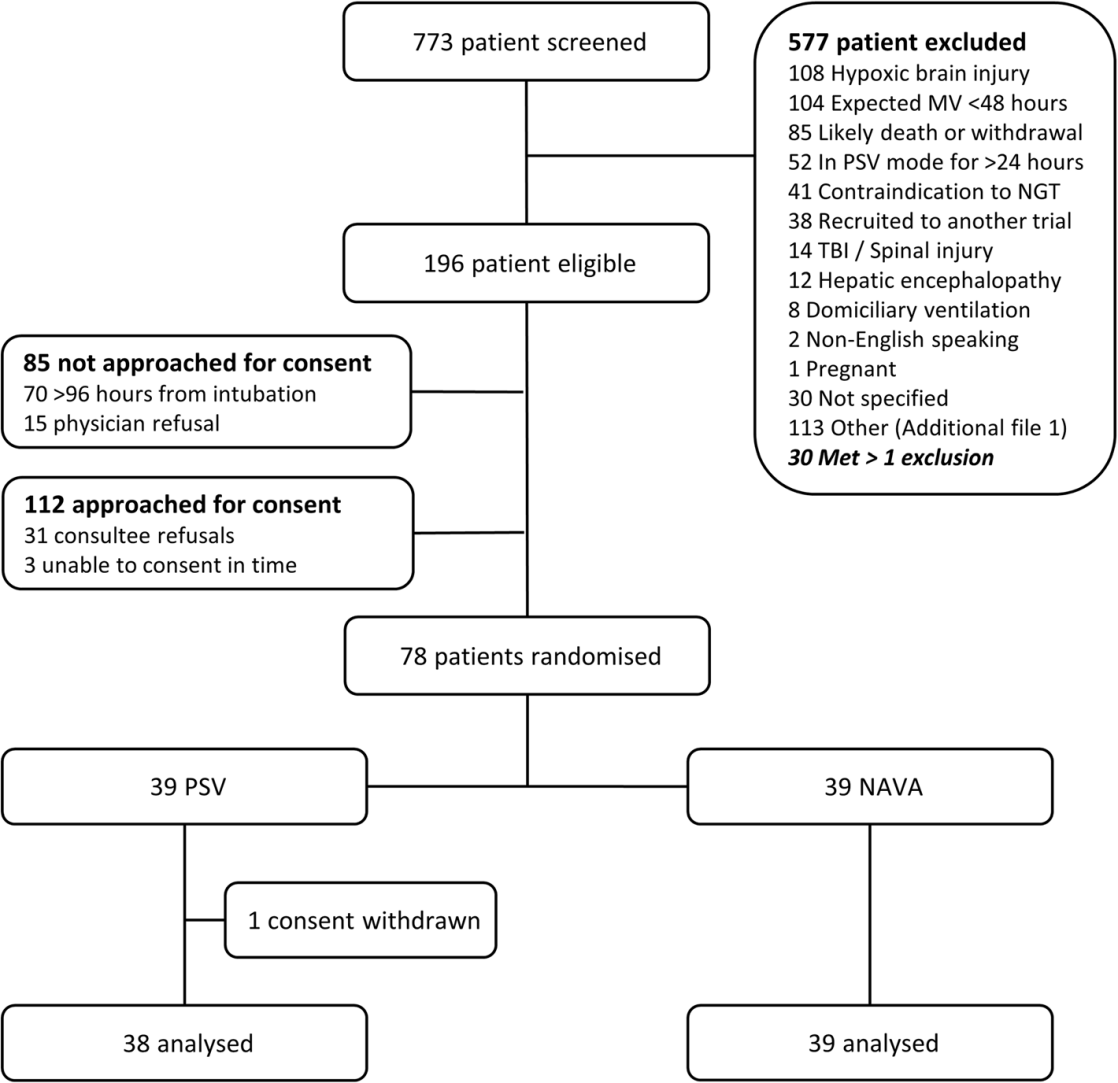
Flow diagram of the clinical trial. NAVA = neurally adjusted 155 ventilatory assist; PSV = pressure support ventilation; NGT = naso-gastric tube; MV = mechanical ventilation; TBI = traumatic brain injury

Over a 45-month enrolment period, 774 invasively ventilated patients with COPD, HF, or ARDS were identified, 112 eligible patients were approached, and 78 participants (39 NAVA and 39 PSV) were recruited, a consent rate of 72% (3 patients consented outside the recruitment window and were subsequently excluded) (Fig. 1). Baseline characteristics of the two groups were similar (Table 1) with good balance across groups for COPD (stratification factor), HF, and ARDS diagnoses. Median durations of MV in any mode and in PSV before randomization were similar (Table 1).

**Table 1:** Baseline characteristics

Variable	NAVA (*n* = 39)	PSV (*n* = 38)
Age, year (mean, SD)	66.7 (13.9)	67.1 (12.9)
Males, *n* (%)	26 (66.7)	28 (73.7)
BMI, kg/m^2^ (mean, SD)	26.5 (4.7)	26.4 (5.7)
Duration of IMV pre-randomization, days (median, IQR)	1.7 (1.1–3.1)	1.7 (0.7–3.0)
Duration of PSV pre-randomization, h (median, IQR)	0.0 (0.0–4.0)	0.0 (0.0–0.3)
APACHE II (mean, SD)	20.5 (6.0)	20.1 (6.1)
SOFA (median, IQR)	8.0 (6.0–8.0)	8.0 (5.5–10.0)
PaO_2_/FiO_2_ ratio, mmHg (mean, SD)	227.0 (82.0)	242.0 (83.0)
PEEP, cmH_2_0 (mean, SD)	8.9 (2.7)	8.9 (2.8)
RASS (median, IQR)	− 3.0 (− 4.0 to − 3.0)	− 4.0 (− 4.3 to − 3.0)
Surgical admission, *n* (%)	13 (33.3)	17 (44.7)
COPD, *n* (%)	14 (35.9)	13 (34.2)
Heart failure, *n* (%)	25 (64.1)	26 (68.4)
Acute respiratory distress syndrome, *n* (%)	6 (15.4)	8 (21.1)
Primary ICU admission diagnosis, *n* (%)		
Cardiovascular	23 (59.0)	20 (52.6)
Respiratory	9 (23.1)	13 (34.2)
Sepsis	4 (10.3)	3 (7.9)
Other^a^	3 (7.7)	2 (5.3)
Comorbidities, *n* (%)		
Chronic lung disease	17 (43.6)	16 (42.1)
Heart disease	24 (61.5)	28 (73.7)
Other^b^	25 (64.1)	22 (57.9)
Current smoker, *n* (%)	6 (15.4)	6 (15.8)
Co-interventions at randomization, *n* (%)		
Continuous inotrope or vasopressor infusion	29 (74.1)	31 (81.6)
Continuous sedation infusion	36 (92.3)	34 (89.5)
Continuous opioid infusion	38 (97.4)	37 (97.4)
Enteral or parenteral nutrition	26 (66.7)	26 (68.4)
Antibiotics	35 (89.7)	35 (92.1)
Invasive cardiac output monitoring	19 (48.7)	21 (55.3)
CRRT/dialysis	13 (33.3)	19 (50.0)

*BMI* body mass index, *APACHE II* Acute Physiology and Chronic Health Evaluation, *COPD* chronic obstructive pulmonary disease, *IMV* invasive mechanical ventilation, *SOFA* Sequential Organ Failure Assessment, *MV* mechanical ventilation, *CRRT* continuous renal replacement therapy, *SD* standard deviation

^a^Gastrointestinal, neurologic, trauma, metabolic

^b^Diabetes, chronic liver disease, neuromuscular disorder (peripheral and/or central), solid organ malignancy, immunosuppression, chronic renal impairment, haematologic malignancy, psychiatric disorder, aids/HIV

### Feasibility outcomes

The average recruitment rate was 1.7 patients per month. Low rates of physician enrolment refusal (15 patients, 11.8%) (Fig. 1) suggests the trial was acceptable to clinical staff. Of the 78 randomized participants, one withdrew consent and five (three NAVA, two PSV) did not commence weaning and were excluded from the adherence analysis. In the 72 participants in whom weaning was attempted, median (95% CI) proportion of time in assigned ventilator mode from randomization to extubation, death, or D28 was 83.1% (64.0 to 97.1%) (NAVA) and 100% (100 to 100%) (PSV). Compliance, i.e. ≥ 65% adherence to assigned mode, was met in 82.2% (95% CI 71.7 to 89.4%) of all participants, 66.7% of NAVA participants (95% CI 50.3 to 80.0%), and 100% of PSV participants (95% CI 89.0 to 100.0%). Mode cross-over occurred in 28 (71.8%) NAVA and 3 (8.3%) PSV participants; the proportion of time cross-over from randomization to extubation, death, or D28 was 17.7% (95% CI 3.2 to 51.4%) and 0% (95% CI 0 to 0%) respectively (Table 2).

**Table 2:** Feasibility outcomes

Variable	NAVA (*n* = 36)^a^	PSV (*n* = 36)^a^
Assigned mode adherence, % (median, 95% CI)^b^	83.1 (64.0–97.1)	100.0 (100.0–100.0)
Compliant participants (≥ 65% adherence), *n* (%, 95% CI)^c^	24 (66.7, 50.3–80.0)	36 (100.0, 89.0–100.0)
Proportion of time cross-over, % (median, 95% CI)^c^	16.9% (2.9–37.5)	0.0 (0.0–0.0)
Total time in NAVA, h (median, IQR)	42.5 (4.5–150.5)	0.0 (0.0–0.0)
Total time in PSV, h (median, IQR)	12.0 (2.0–29.0)	89.0 (13–185.5)
Participants with cross-over, *n* (%)	28 (77.8)	3 (8.1)
Reasons for cross-over, *n* (%)^d^		
Edi signal problem	10 (27.8)	–
Edi signal noise or interference	8 (22.2)	–
Low or absent Edi	2 (5.6)	–
Clinical rationale/clinical team preference	11 (30.6)	3 (8.3)
General instability or deterioration	7 (19.4)	1 (2.8)
Tachypnoea	1 (2.8)	–
Ventilator dyssynchrony	1 (2.8)	3 (8.3)
Lack of trial awareness	5 (13.9)	–
Clinical inexperience with NAVA	4 (11.1)	–
NAVA tube insertion difficulty	2 (5.6)	–
NAVA catheter removed for tor transfer	1 (2.8)	–
Not documented	8 (22.2)	–
Participants with > 1 reason for cross-over	8 (22.2)	–

*NAVA* neurally adjusted ventilatory assist, *PSV* pressure support ventilation, *IQR* interquartile range, *Edi* electrical activity of the diaphragm, *CI* confidence interval

^a^Three NAVA arm participants and 2 PSV arm participants did not use either NAVA or PSV and were therefore excluded

^b^Proportion of time in assigned mode = time in the mode assigned at randomization (NAVA or PSV) as a proportion of total time in continuous, spontaneously triggered ventilation modes, either PSV or NAVA. Crossed over hours due to initial set-up (within 4 h of randomization) or SBT (PSV ≤ 5 cm H_2_O) were permitted in the protocol and discounted from this calculation

^c^Compliance = ≥ 65% adherence to the ventilation mode assigned at randomization

^d^Participants may experience > 1 reason for cross-over; therefore, the sum of percentages is not 100%

The main reasons for crossover in the NAVA arm were Edi signal noise or interference causing loss of synchrony (8/28, 22.2%), clinical team preference for use of PSV during deterioration or instability (7/28, 19.4%), lack of trial awareness (5/28, 3.6%), and clinical inexperience leading to delayed application of NAVA (4/28, 14.3%). The three cross-overs in the PSV arm were due to perceived ventilator dyssynchrony (Table 2).

### Exploratory clinical outcomes

**Fig. 2:**
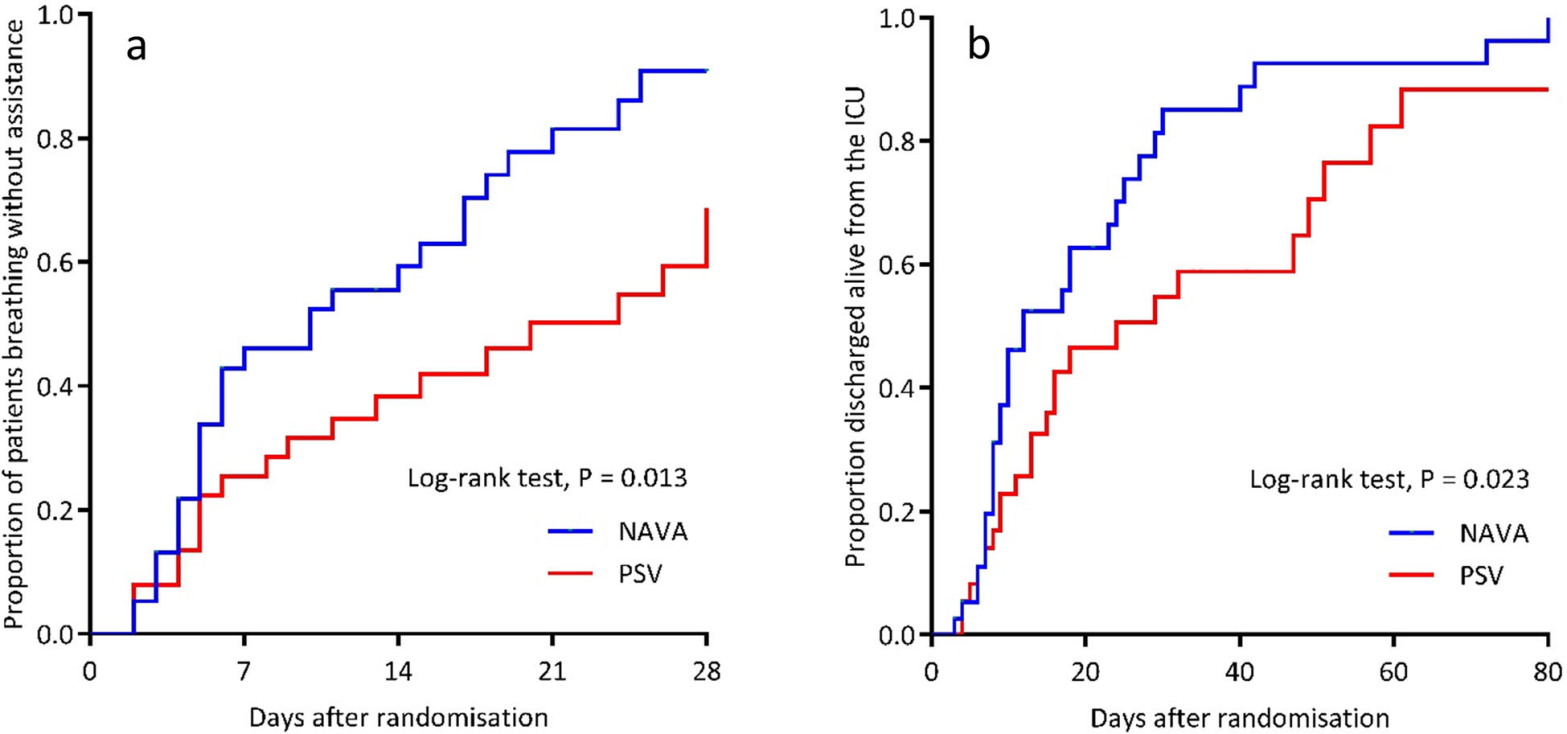
Kaplan-Meier estimates of probability of unassisted breathing and live discharge from ICU from randomization to D28. In keeping with previous trials [21, 22], unassisted breathing is defined as (1) extubated with supplemental oxygen or room air, or (2) open T-tube breathing, or (3) tracheostomy mask breathing, or (4) continuous positive airway pressure (CPAP) ≤ 5 cm H_2_0 without pressure support and with no return to assisted breathing or death within 48 h. Participants receiving pressure support via non-invasive ventilation or CPAP > 5 cm H_2_O via any medium were defined as receiving assisted ventilation. NAVA = neurally adjusted ventilatory assist; PSV = pressure support ventilation

Median (IQR) VFDs to D28 were greater in NAVA with a median difference of 3.0 VFDs (95% CI to 0.011.0; *p* = 0.04), 15.5 VFDs in NAVA (0.0 to 23.0) compared to 0 VFDs (0.0 to 20.5) in PSV (Table 3). The median (IQR) time to first extubation was 3.7 days (1.9 to 4.9) (NAVA) versus 4.4 days (1.9 to 7.9) (PSV) (MD 1.0 day, 95% CI 0.8 to 3.0; *p* = 0.23). The overall median (IQR) duration of MV was 4.9 days (2.8 to 15.7) (NAVA) versus 9.8 days (3.6 to 25.9) (PSV) (MD 3.0 days, 95% CI 0.4 to 8.6; *p* = 0.13). Time to breathing without ventilator assistance and time to alive ICU discharge were shorter in NAVA (log-rank tests, *p* = 0.01 and *p* = 0.02 respectively, Fig. 2). Hospital mortality was lower in the NAVA group (9 deaths, 23.1%) compared to the PSV group (19 deaths, 50.0%) (RR 0.5, 95% CI 0.2 to 0.9; *p* = 0.032). No significant difference was observed in ICU mortality (8 deaths [20.5%] versus 15 deaths [39.5%]; *p* = 0.085), 28-day mortality (8 deaths [20.5%] versus 11 deaths [28.9%]; *p* = 0.44), and D90 mortality (9 deaths [24.3%] versus 17 deaths [44.7%]; *p* = 0.09) (Table 3) in the NAVA and PSV groups respectively (Table 3).

**Table 3:** Secondary outcomes

Variable	NAVA (*n* = 39)	PSV (*n* = 38)	Effect estimates NAVA–PSV	*P* value
Ventilator-free days, days (median, IQR) ^a^			***MD (95% CI)***	
From randomization to D28	15.5 (0.0–23.0)	0.0 (0.0–20.5)	3.0 (0.0–11.0)	0.041
From randomization to D90	75.5 (36.0–85.0)	35.5 (0–82.5)	8.0 (0.0–48.0)	0.036
Time to first extubation, days (median, IQR), *n*	3.7 (1.9–4.9), 25	4.4 (1.9–7.9), 26	− 1.0 (− 3.0–0.8)	0.228
Time to successful extubation, days (median, IQR), *n*^a^	4.2 (1.7–8.7), 23	3.9 (2.3–7.9), 20	0.1 (− 2.2–2.1)	0.957
Duration of MV, days (median, IQR)^a^	4.9 (2.8–15.7)	9.8 (3.6–106.3)	− 3.0 (− 8.6–0.4)	0.094
Combined CMV mode hours, (median, IQR), *n*	61.0 (33.0–118.0), 35	69.0 (38.0–128.0), 37	− 6.0 (− 33.0–22.0)	0.604
Duration of NIV to D28, h (median, IQR), *n*	23.0 (9.5–106.3), 4	28.0 (5.0–41.0), 8	− 0.5 (− 24.0–106.0)	> 0.999
ICU stay, days (median, IQR)	9.1 (6.0–21.9)	14.8 (7.0–33.1)	− 3.3(− 8.8–1.1)	0.158
Hospital stay, days (median, IQR)	19.9 (11.9–42.8)	26.6 (11.3–61.1)	− 4.0 (− 14.9–5.0)	0.419
			***RR (95% CI)***	
NIV within 48 h post-extubation, *n* (%)	4/25 (16.0)	6/26 (23.1)	0.7 (0.2–2.0)	0.727
ICU mortality, *n* (%)	8/39 (20.5)	15/38 (39.5)	0.5 (0.3–1.1)	0.085
Hospital mortality, *n* (%)	9/39 (23.1)	19/38 (50.0)	0.5 (0.2–0.9)	0.032
Ventilator-associated pneumonia, *n* (%)	12/39 (30.7)	10/38 (26.3)	1.2 (0.6–2.4)	0.802
Pneumothorax, *n* (%)	1/39 (2.6)	0/38 (0.0)	1.2 (0.6–72.4)	0.801
Self extubation, *n* (%)	3/39 (7.7)	2/38 (5.3)	1.5 (0.3–7.1)	> 0.999
Participants with failed extubation, *n* (%)	6/25 (24.0)	7/26 (26.9)	0.9 (0.4–2.2)	> 0.999
Participants with reintubation, *n* (%)	7/25 (28.0)	9/26 (34.6)	0.8 (0.4–1.8)	0.765
Tracheostomy, *n* (%)	10/39 (25.6)	11/38 (29.0)	0.9 (0.4–1.8)	0.745

All durations measured from randomization

*NAVA* neurally adjusted ventilatory assist; *PSV* pressure support ventilation; *IQR* interquartile range; *NIV* non-invasive ventilation defined as either non-invasive positive pressure ventilation (NIPPV) or continuous positive airways pressure (CPAP) > 5 cm H_2_O; *MV* mechanical ventilation defined as any ventilation support via an endotracheal tube, or tracheal or non-invasive ventilation > 5 cm H_2_0 of CPAP; *MD* median difference, calculated using the Hodges Lehmann estimating method [15]; *RR* relative risk

^a^Excludes participant 76 who had prior home dependence on bi-level ventilation

Mean hourly RASS scores were closer to zero in the NAVA mode (RASS recorded in NAVA mode in the NAVA arm: − 0.5 [− 1.6 to − 0.1], *n* = 33; compared to RASS recorded in PSV mode in the PSV arm: − 1.4 [− 2.5 to − 0.8], *n* = 35; MD − 0.8, 95% CI − 1.4 to − 0.1; *p* = 0.020), indicating less sedation while ventilated using NAVA (Table 4). However, no difference between the groups was observed in mean RASS while invasively ventilated including all modes, sedative, or analgesic infusion doses by infusion day, or in the mean number of bolus doses administered per day (Table 4). Incidence of VAP and self/unplanned extubation were similar between groups (Table 3). There were no other adverse events recorded suggesting the trial protocol was safe.

**Table 4:** Sedation outcomes

Variable	NAVA	PSV	Effect estimates NAVA-PSV MD (95% CI)	*P* value
Propofol dose, g (median, IQR), *n*^a^	1.7 (1.1 to 2.6), 32	1.8 (1.0 to 2.8), 33	0.0 (− 0.6 to 0.5)	0.933
Midazolam dose, mg (median, IQR), *n*^a^	50.3 (29.3 to 84.9), 14	54.0 (26.0 to 95.3), 15	− 3.3 (− 41.5 to 25.5)	0.706
Fentanyl equivalents dose, mg (median, IQR), *n*^a,b^	2.1 (1.3 to 3.4), 39	2.2 (1.6 to 3.5), 36	− 0.1 (− 0.7 to 0.5)	0.706
Bolus doses, *n* (median, IQR)^a^	2.4 (0.7 to 4.2)	2.7 (1.6 to 4.3)	0.0 (− 1.3 to 1.0)	0.902
RASS while invasively ventilated (median, IQR)	− 2.1 (− 3 to − 0.9)	− 2.4 (− 3.6 to − 1.5)	0.4 (− 0.2 to 1.0)	0.139
RASS while in assigned mode (median, IQR), *n*^c^	− 0.5 (− 1.6 to − 0.1), 33	− 1.4 (− 2.5 to − 0.8), 35	0.8 (0.1 to 1.4)	0.020
RASS in CMV modes (median, IQR), *n*^d^	− 3.0 (− 3.7 to − 2.5), 35	− 3.5 (− 4.0 to − 2.8), 37	0.4 (0.0 to 0.9)	0.059

Doses are mean total administered dose per infusion day from randomization to study end. Bolus doses are mean number administered per infusion day. RASS assessed and recorded hourly by clinical staff and mean scores were calculated for each participant; group medians of individual participant mean scores were analysed

*IQR* interquartile range, *RASS* Richmond Agitation and Sedation Scale, *NMB* neuromuscular blockers, *MD* median difference, calculated using the Hodges Lehmann estimating method [15]

^a^Excludes participant 20. Source data missing

^b^Fentanyl equivalent conversion factors: 1 mg fentanyl = 0.5 mg, remifentanyl = 100 mg, morphine = 50 mg diamorphine

^c^Six NAVA arm and 2 PSV arm participants did not receive the assigned mode. RASS scores were missing in one PSV arm participant

^d^Two NAVA arm participants did not receive CMV

## Discussion

This four ICU, single-centre, parallel group, RCT has demonstrated protocol feasibility by achieving acceptable adherence to the assigned mode and an acceptable proportion of compliant patients. The study has also demonstrated protocol acceptability by low rates of physician recruitment refusal and high participant consent rates and protocol safety via the absence of adverse events. Acknowledging the possibility of chance findings in this relatively small study, the exploratory clinical outcomes suggest increased VFDs, reduced time to breathing without assistance and reduced time to ICU discharge in the NAVA group, and improved sedation management while in the NAVA mode. These feasibility data improve our understanding of NAVA compared to PSV beyond 48 h of application, improving the chances of success in any subsequent trial. The clinical results are consistent with those reported by Demoule et al. [3] and suggest that a fully powered trial is justified.

Despite satisfactory mode adherence and protocol compliance across groups, these proportions were lower in the NAVA group, and substantial (albeit for a short duration) cross-over from NAVA was observed. Of the reported reasons for cross-over, human factors such as trial awareness, clinician preference, and lack of NAVA experience were most common, implying low confidence in the application of NAVA and need for further training. These issues may have been expected in a large group of clinical staff (over 300) despite attempts to embed NAVA technology into unit practice. This finding also highlights the complexity of NAVA and the likely variation in clinical application caused by ICU contexts and human interactions.

While the influence of human factors described above may be modified with increased/improved training and changes to study documents, of more concern for trial feasibility is the difficulty in acquiring and maintaining a satisfactory Edi signal, which occurred in 10 out of 36 (27.8%) NAVA participants. Edi signal problems have been reported previously. Di Mussi et al. reported NAVA mode failure in seven out of 20 patients (35%) due to loss of ‘Edi synchrony’ or low Edi activity, despite having obtained a reliable Edi signal at baseline [23]. Demoule et al. reported high levels of mode adherence across the first 48 h in NAVA (median 44.1 h [IQR 33 to 47.8]), but do not report on cross-over or its reasons. Recruitment after successful commencement of PSV in the study by Demoule et al. may be relevant. However, the reasons for the disparities between these trials and for the Edi difficulties described remain unclear. Varying levels of clinical expertise across a large staff group may be expected and relevant, but the results may also suggest limitations in NAVA technology (including anatomical abnormalities such as hiatal hernia) and/or a technical complexity in its clinical application over and above those associated with PSV. Notably, both trials were conducted in academic centres; therefore, these issues may be further magnified in non-academic centres with less experience of MV and NAVA.

In contrast to previous studies, the exploratory analysis of ventilation and clinical outcomes found potential benefit in the NAVA arm with increased VFDs to D28, decreased time to breathing without assistance and to alive ICU discharge, and reduced hospital mortality. Acknowledging the low power of these analyses, certain characteristics of our study that are different to previous studies may be relevant to the interpretation of these results. We specifically selected patients with risk factors for prolonged MV [12] and conditions where NAVA has potential physiological benefits [24–26]. In addition, Edi monitoring was used in the NAVA arm only, meaning the trial assessed the combined effects of monitoring and NAVA. Neural respiratory drive (Edi) monitoring is a potential advantage of NAVA [27] as it may encourage adjustment and improvement of ventilator settings, decrease sedative use, and expedite clinical assessment of weaning readiness. Clinical factors such as renal replacement therapy for example, which differed between the groups, or the lack of an extubation protocol, may also be of relevance to the results. Accepting the need for cautious interpretation, outcomes favouring NAVA are consistent with most published physiological data on NAVA and with trends toward superior clinical outcomes in two previous NAVA RCTs [3, 4]. As such, our findings strengthen the support of a future definitive, adequately powered RCT.

In addition to benefits in ventilation and clinical outcomes and despite no difference in sedation dose and RASS during in all MV modes, RASS scores were closer to zero in the NAVA mode compared to the PSV mode (*p* = 0.020), potentially indicating improved sedation management. Decreases in sedation may be driven by the need to optimise the Edi signal and ensure optimal levels of respiratory drive are present prior to the initiation of NAVA [28, 29]. Improved synchrony and patient comfort when in the NAVA mode may further reduce sedative requirements. Reduced sedation [30] and improved COMFORT scores [31–33] have been suggested in paediatric trials, but there are limited data available from adult studies. Demoule et al. [3] showed improved dyspnoea at day 1, but no overall difference in adaptation to intensive care environment (ATICE) comfort scores [34] in patients ventilated with NAVA versus PSV. Coisel et al. [35] found no difference in RASS in a small randomized cross-over study in 14 patients ventilated post-operatively. However, while sedation load and RASS outcomes may provide insight into the potential mechanisms underlying beneficial effects of NAVA, the individual clinical relevance and relative clinical value remain unclear.

### Strengths and limitations

A key strength of this study is the description of mode adherence and reasons for poor adherence over prolonged durations. The use of a pragmatic protocol ensures that these adherence data are relevant to the real-world application of NAVA. The study successfully recruited patients at risk of prolonged MV, and treatment separation was improved due to the application of Edi monitoring in the NAVA arm only. While early recruitment during acute critical illness allowed assessment of Edi in CMV modes and NAVA from the very start of spontaneous breathing, it also led to the inclusion of some patients who did not commence weaning and potentially increased the rate of short-term NAVA failure during efforts to commence weaning.

Despite conducting the trial in four distinct units, the interpretation of the results and the degree to which the results are generalizable is limited due to its relatively small size and conduct at a single institution. As in the Demoule trial, the study site is an academic centre with relatively high levels of MV experience, which may limit the generalisability of results to less experienced centres. NAVA was introduced 5 years prior to the start of the trial in 2008; approximately 50% of staff were trained and 70% had clinical experience of NAVA. NAVA training and experience was similar across the four ICUs.

An analysis of ventilator synchronisation was beyond the scope of this study, and the absence of these data may further limit the interpretation of the exploratory outcomes. Improved synchrony in NAVA compared to PSV has, however, long been established [3, 4, 23, 28, 35–41], and dysynchrony is recognised as being associated with worse patient outcomes [42, 43]. As is common to many MV studies, blinding the clinical team was not possible. Outcome assessors and data analyses were also unblinded, although ventilator downloads verified objective outcomes, partially limiting the potential for bias.

A further potential source of error in the determination of NAVA compliance is the rate of automatic switching to PSV as a NAVA back-up mode, which was not possible to capture during the current trial. Although previous reports suggest levels of automatic switching to PSV of only 0.5 to 2% of total time in NAVA [3, 33], it remains a concern when considering the effect on clinical outcomes being attributed to NAVA ventilation in the current study.

As discussed above, NAVA is a complex intervention and its application will vary with human interactions and ICU context. Therefore, to enable interpretation of a multi-site trial a process, a process evaluation will be essential to determine whether the intervention was delivered as intended and to provide descriptive information relating to the research context [44].

## Conclusions

This study is the first to our knowledge to report NAVA mode adherence rates beyond 48 h and reasons for cross-over, methodological evidence which is key to the success of future clinical trials. Together with the clinical benefit suggested in secondary outcomes, this study suggests that it is both feasible and justified to conduct a definitive randomized controlled trial to establish the effectiveness of NAVA in patients with a high likelihood of prolonged MV.

## Supplementary information

**Additional file 1.** Additional methods information.

**Additional file 2.** Supplemental data tables.

## Data Availability

The datasets generated analysed during the current study are available from the corresponding author on reasonable request.

## Abbreviations

APACHE: Acute Physiology and Chronic Health Evaluation
ARDS: Acute respiratory distress syndrome
CRRT: Continuous renal replacement therapy
CI: Confidence interval
CMV: Continuous mandatory ventilation
COPD: Chronic obstructive pulmonary disease
CPAP: Continuous positive airway pressure
Edi: Electrical activity of the diaphragm
HF: Heart failure
ICU: Intensive care unit
IQR: Intra-quartile range
MD: Median difference
MV: Mechanical ventilation
NAVA: Neurally adjusted ventilatory assist
PEEP: Positive end expiratory pressure
PSV: Pressure support ventilation
RASS: Richmond Agitation and Sedation Scale
RCT: Randomised controlled trial
RR: Relative risk
SD: Standard deviation
VAP: Ventilator-associated pneumonia
VFDs: Ventilator-free days
