# Supplementary material for: Neurally adjusted ventilatory assist versus pressure support ventilation: a randomized controlled feasibility trial performed in patients at risk of prolonged mechanical ventilation
Source: Crit Care. 2020 May 14;24:220. doi: 10.1186/s13054-020-02923-5 (PMC7224141; doi:10.1186/s13054-020-02923-5)
Supplement: Supplementary file 1 — Additional file 1. Additional methods information. [file 13054_2020_2923_MOESM1_ESM.docx]

**Additional file 1: Methods**

**Article title**

Neurally Adjusted Ventilatory Assist versus Pressure Support Ventilation: A Randomized Controlled Feasibility Trial

**Authors**

Daniel J. Hadfield ^1,2^, Louise Rose ^3,4^, Fiona Reid ^5^, Victoria Cornelius ^6^, Nicholas Hart ^2,7^, Clare Finney ^1^, Bethany Penhaligon ^1^, Jasmine Molai ^1^, Clair Harris ^1^, Sian Saha ^1^_,_ Harriet Noble ^1^, Emma Clarey ^1^, Leah Thompson ^1^, John Smith ^1^, Lucy Johnson ^1^, Phillip A. Hopkins ^1^, Gerrard F. Rafferty ^2^

^1^ Critical Care, King's College Hospital, London, United Kingdom

^2^ Centre for Human and Applied Physiological Sciences, King's College London, United Kingdom

^3^ Florence Nightingale Faculty of Nursing, Midwifery & Palliative Care, King’s College London, United Kingdom

^4^ Sunnybrook Health Sciences Centre and Sunnybrook Research Institute, Toronto, Canada

^5^ School of Population Health and Environmental Sciences, King's College London, London, United Kingdom

^6^ Faculty of Medicine, School of Public Health, Imperial College

^7^ Lane Fox Unit, Guy's and St Thomas' NHS Foundation Trust, London, England

**Corresponding Author:**

Daniel Hadfield. Email: daniel.hadfield@nhs.net.

**Contents**

[1. Participants 3](#_Toc34216596)

[2. Randomization 4](#_Toc34216597)

[3. Statistics 4](#_Toc34216598)

[4. Procedures 5](#_Toc34216599)

[4.1. Neurally Adjusted Ventilatory Assist (NAVA) catheter insertion and positioning 6](#_Toc34216600)

[4.2. Edi monitoring 7](#_Toc34216601)

[4.3. Commencement and setting of MV 7](#_Toc34216602)

[4.4. Troubleshooting 10](#_Toc34216603)

[5. Weaning recommendations 10](#_Toc34216604)

[6. Sedation holds and spontaneous breathing trials 11](#_Toc34216605)

[7. Sedation 12](#_Toc34216606)

[8. Education and optimisation of compliance 13](#_Toc34216607)

[9. Monitoring and data recording 13](#_Toc34216608)

[10. Outcomes 14](#_Toc34216609)

[12. References 19](#_Toc34216610)

The following document provides methodological information to supplement the main manuscript. A summary protocol is available on www.clinicaltrials.gov (NCT01826890)

# Participants

The population of interest were intubated and ventilated adult patients at risk of difficult or prolonged weaning: Those with pre-existing cardiopulmonary dysfunction [1]. All adult Intensive Care Unit (ICU) admissions to the four participating ICUs were screened to identify those receiving invasive mechanical ventilation (MV) expected to continue for >48 hours with at least one of the following risk factors for difficult or prolonged weaning [1]: (1) chronic obstructive pulmonary disease (COPD), (2) heart failure (HF); or (3) mild, moderate of severe acute respiratory distress syndrome (ARDS) defined according to Berlin criteria [2]. A diagnosis of COPD, HF or ARDS was determined by research staff through documentation in the medical record by senior grade specialist physician (respiratory, cardiac, intensivist) or a non-specialist physician diagnosis combined with either objective test results (spirometry, a CT scan, lung biopsy, cardiac echocardiogram) and/or prescribed treatment. A confirmed diagnosis documented according to these criteria was required prior to study entry. Post randomization, HF was confirmed using N-terminal pro b-type natriuretic peptide (NT-proBNP) and Troponin tests.

Exclusion criteria were: likely extubation, death, or treatment withdrawal within 48 hours confirmed with the treating medical team; >96 hours from intubation; >24 hours of Pressure Support Ventilation (PSV), NAVA or any other continuous spontaneous ventilation mode; suspected or proven hypoxic brain injury; high spinal injury above C6; severe traumatic brain injury with Glasgow Coma Score < 8; neurological cause of ventilator dependency such as Guillain-Barré syndrome or Myasthenia Gravis; contraindication to nasogastric tube insertion; requirement for domiciliary ventilation with the exception of that used for sleep disordered breathing only; enrolment in any other interventional clinical trial; non-English speakers where inadequate translation available to allow informed consent; and pregnancy.

# Randomization

All participants were sedated and intubated, therefore informed consent was obtained from legal surrogates. Participants were randomized with allocation concealment using online software developed and managed by an independent Clinical Trials Unit (King’s College London Clinical Trials Unit) [3]. Permuted block randomization with random block sizes, stratified for COPD were used. The trial had an open-label design due to inability to blind the study intervention, with treating clinicians, researchers, participants, families and outcome assessors aware of the study-group allocation.

# Statistics

The central aim of the study was to investigate feasibility; therefore a formal power calculation was not required. A confidence interval approach was used to determine the number of participants needed to provide reasonable precision of the primary feasibility parameter, protocol compliance [4]. Using 65% as the lower bound of the confidence interval (the minimum acceptable proportion of compliant participants that would indicate feasibility) and an expected attrition of 5% (where a patient does not use NAVA or PSV in the study period), a sample size of 76 patients (38 in each arm) would estimate an anticipated compliance rate of 75% to within a 95% confidence interval of +/- 10% [5]. This calculation included anticipated attrition due to non-use of either NAVA or PSV during the 28-day period from randomisation, either due to severity of illness, death, or the possibility of successful extubation from a mandatory mode.

Descriptive statistics were used to characterize the sample. Categorical data using chi-square or Fisher exact tests and continuous variables using non-parametric Mann–Whitney tests or independent samples t-tests as appropriate. Effect sizes in continuous variables are reported as median difference (MD) using the Hodges Lehmann estimating method [6], and estimates of effect in binary data as relative risk (RR) [7]. 95% CIs are reported for compliance and effect estimates [7, 8]. Time to breathing without assistance and live ICU discharge were displayed using Kaplan-Meier curves with log-rank tests for significance. A two-sided P value of <0.05 was considered to be statistically significant. Qualitative descriptions of cross-over were categorized using content analysis [9]. Statistical analyses were performed using GraphPad Prism version 7.04 for Windows (GraphPad Software, La Jolla California USA).

# Procedures

All participants were ventilated using Servo-i®™ or Servo-u®™ ventilators (Getinge, Solna, Sweden). The study site is a large, multi-speciality academic teaching hospital; PSV is the long-established main mode for weaning and NAVA was introduced in 2008, five years prior to the start of the current study. The study protocol initially advised the placement of NAVA catheters in all patients. However, ethical approval was obtained to withdraw this requirement from PSV participants. This was in response to feedback from some clinicians, who preferred to avoid the risk of NG catheter replacement in sicker patients, where the NAVA catheter would be used for data collection only and not for NAVA ventilation. It was hoped that this would help with recruitment rate. In total, 16/38 (42.1%) of PSV participants received a NAVA catheter. In these PSV arm participants, NAVA catheters were connected intermittently by research staff for data capture with the Edi signals blinded to clinical staff. Cables were disconnected, and Edi signals blinded at all other times.

In the NAVA arm, NAVA catheters were inserted, and participants had continuous monitoring of diaphragmatic electrical activity (Edi) and use of the NAVA mode in place of PSV. In the PSV group, participants were ventilated according to usual local practice. The study intervention in both arms was continued for a maximum of 28-days from randomization, after which point clinicians were free to control the ventilation mode. Protocols and practice recommendations were included within bedside study packs to guide clinical staff in the management of MV and sedation. Adherence to these protocols was not mandated; clinical decisions including the indications for and timing of spontaneous MV modes, remained at the discretion of the clinical team. Compliance was optimized using bedside study packs and posters, and daily reminders from research staff. Group and individual education sessions were provided throughout the by study investigators.

Clinical need took precedent over study interventions. MV settings were only changed with the full agreement of the ICU consultant in charge of the participant’s care, and NAVA was only initiated when the clinical team agreed that the patient was able to tolerate a spontaneous MV mode. In all participants, clinicians were advised to use protective MV settings, targeting tidal volumes of between six and eight millilitres per kilogram of predicted body weight [10]. The following information was provided within study specific protocols and guidelines, placed within the bedside study packs. Protocols were produced with reference to the manufacturer guidelines (Getinge, Solna, Sweden) and with informal guidance from local and international experts in MV. Adherence to these protocols was not mandated; clinical decisions including the indications for and timing of spontaneous MV modes, remained at the discretion of the clinical team.

## Neurally Adjusted Ventilatory Assist (NAVA) catheter insertion and positioning

In NAVA arm participants, NAVA catheters were inserted and positioned within four hours of randomization by trained clinical or research staff according to manufacturer guidelines and according to local guidelines for safe placement and use of naso-gastric (NG) catheters. With the agreement of the treating clinical team, existing catheters were removed. Where NAVA catheters were present or subsequently inserted in PSV group patients, viewing of the Edi signal and use of the NAVA mode was not be permitted; NAVA technology was disabled (NAVA modules removed)

Once inserted, the catheter was connected to the ventilator and positioning was achieved using a dedicated positioning tool on the Getinge® ventilator interface, which enabled optimum positioning of the electrodes relative to the diaphragm. The catheter position was checked daily and recorded by research and clinical staff using the catheter-positioning tool and manufacturer guidelines. Incidence of catheter migration / repositioning were recorded.

## Edi monitoring

This guidance was applied when there was no need for continuous mandatory ventilation (CMV) or deep sedation with Richmond Agitation and Sedation Scale (RASS) score <-2. On an hourly basis, the bedside nurse observed and recorded the maximum Edi on the observation chart. The figure recorded was an estimated average of at least three breaths (current standard local practice). A lower target level of 8µV was set following basic analysis of existing literature and local audit data to avoid over-ventilation and over-sedation. Current locally written sedation and ventilation guidelines were followed in all patients. In summary:

- Target a RASS score of -2 to 0 by adjustment of sedation (local guidelines)
- Target an Edi of >/= 8µV by adjustment of sedation dose and/or adjustment of MV settings where appropriate
- Record the maximum Edi on the observation chart hourly (estimated average of at least three breaths)
- Note the trend of the Edi signal on each patient assessment

## Commencement and setting of MV

In all participants, clinicians were advised to use protective MV settings, targeting tidal volumes of between six and eight millilitres per kilogram of predicted body weight [10]. In both arms, Spontaneous Breathing Trials (SBTs) and sedation holds were recommended daily practice while the participants were weaned from MV support. Study specific protocols were designed and included in bedside patient packs. MV settings were altered by trained clinical or research staff in coordination with the ICU team. At the earliest possible opportunity following randomization and catheter placement, when there was evidence of spontaneous breaths, spontaneous mode assessments were performed in either NAVA or PSV (depending on randomization) using the process described below:

Eligibility and safety screens were performed by research or clinical staff. Patients were eligible if all the following applied:

- Current use of invasive MV and no requirement for CMV
- Oxygen saturation ≥88%, fraction of inspired oxygen (FIO_2_) ≤50%, positive end expiratory pressure (PEEP) ≤8 cm H_2_O
- No significant use of vasopressors or inotropes (Dopamine >15 or Epinephrine >0.1 or Norepinephrine >0.1μg/kg/min)
- No current or recent neuromuscular blockers

If eligible, approval was obtained from the treating ICU consultant and the nurse in charge, before MV settings were changed

- Using the ‘NAVA preview’ mode, the NAVA level was adjusted until the expected pressure delivery (a dynamic curve superimposed above actual pressure in the current mode) was equivalent to the actual pressure delivery in the current mode
- Back-up modes (either PSV or Pressure Control modes) were adjusted to suitable levels to guard against the possibility of either under or over support during back-up modes
- PEEP and FiO_2_ remained as per previous settings

At this stage, the NAVA or PSV modes were activated, and support levels and other settings were adjusted to achieve tidal volumes of 6–8 ml/kg of ideal body weight, and according to patient comfort, arterial blood gas results and wider patient assessment. Electrical breath triggering (default = an increase of 0.5 μV above a baseline) and standard pressure or flow triggers were also adjusted as necessary. This process varied for each patient depending on clinical condition, the level of arousal, and the quality of the Edi signal in NAVA arm participants. To raise awareness of synchrony issues and possibilities for adjustments in the PSV arm, clinicians were advised to consider adjusting the cycle-off criteria; to shorten the inspiratory support period and lengthen the ventilator expiratory period, which may be necessary in patients with increased expiratory resistance. They were also asked to consider adjusting the pressure support (PS) velocity to better match patient inspiratory flow demand [11].

Successful completion of a 30-minute period of spontaneous mode breathing was recommended prior to sustained use of the NAVA or PSV modes. The following failure criteria required a return to the previous MV settings:

- Evidence of increased and sustained anxiety (RASS score 2 to 4)
- A sustained drop in O_2_ saturations to less than 88% or a drop of > 5% of baseline (whichever was lowest)
- Heart rate >140 bpm or a change from baseline of 20% in either direction
- An acute cardiac dysrhythmia
- Two or more signs of respiratory distress, including tachypnoea (respiratory rate >20 % from baseline), change in heart rate (as above), new use of accessory muscles, abdominal paradox, new diaphoresis, or marked dyspnoea [12, 13]
- In the NAVA group, an Edi signal that was incompatible with safe, sufficient and synchronised MV (e.g. excessive signal noise or unsustained effort leading to apnoeic periods)

## Troubleshooting

Where there was low or absent Edi signal during catheter positioning, the protocol recommended that clinicians consider potential causes, such as high levels of ventilatory support, high level of sedation, neuromuscular blocking agents, phrenic nerve lesions and myopathy. In the event of low or absent Edi, clinicians were requested to check the catheter positioning, then reduce the ventilatory support and/or sedation and repeat positioning a few minutes later.

# Weaning recommendations

It is understood that despite recent published definitions, weaning practices vary between countries and institutions. For the purposes of this study and based on practice at the study site, weaning was judged to have started at the commencement of a CSV mode, and is simply defined as the entire process of liberating the patient from MV support. Once the NAVA or PSV had been established, the clinical team directed weaning of the support levels (pressure support, PEEP and FiO_2_) with the help of the following guidelines provided in the bedside study pack.

Where there were no signs of distress or ventilatory failure (see above):

1. Gradual stepwise reduction in FIO_2_, PS (e.g. by reduce NAVA level by 0.1 or 0.2 cm/μV or PS levels by 1 or 2cm H_2_O every 2 hours) and PEEP
2. Return to the next higher support level if distress developed or RR>30 breaths/min, allowing at least 2 hours for recovery before recommencement of weaning
3. Weaning to be suspended and optimal support to be maintained from 20:00 hours to 08:00 hours (night time)
4. Daily sedation holds and SBTs where eligible

# Sedation holds and spontaneous breathing trials

Documents recommended that sedation holds and SBTs were conducted daily between 9am and 11am on all research patients up to D28. They were performed by clinical staff or research staff in coordination with the ICU team**.**

Participants were eligible for sedation holds if there was no sedation for the defence of a difficult airway; no sedative infusion for active seizures or alcohol withdrawal; no risk or evidence of increased intracranial pressure; no sedative doses due to uncontrolled agitation; no current or recent neuromuscular blockers; and no evidence of myocardial ischaemia in the previous 24 hours.

All sedatives and analgesics used for sedation were interrupted and analgesics for active pain continued. Patients were monitored for up to four hours and sedation was restarted at half original rates and titrated to achieve light sedation if the following were observed:

- Evidence of increased and sustained anxiety (RASS score 2 to 4).
- O2 saturations drop to less than 88% or a drop of > 5% of baseline (whichever was lowest)
- Heart rate >140 bpm or a change from baseline of 20% in either direction
- An acute cardiac dysrhythmia
- Two or more signs of respiratory distress, including tachypnoea (RR >20 % from baseline), heart rate change (HR >20 % from baseline), new use of accessory muscles, abdominal paradox, new diaphoresis, or marked dyspnoea

Patients using CMV modes were eligible for SBTs if oxygen saturations were ≥88%, with FIO_2_ ≤50% and PEEP ≤8 cm H_2_O, and where there was no uncontrolled agitation, no evidence of myocardial ischaemia in the previous 24 hours, no significant use of vasopressors or inotropes, no risk or evidence of increased intracranial pressure, and no current or recent neuromuscular blockers. Patients were switched to a T-tube circuit or CPAP of ≤5 cm H_2_O +/- PS of ≤5 cm H_2_O, with no change made to FIO_2_. Participants were then closely monitored for 30 minutes for the following failure criteria:

- Increased and sustained anxiety
- A drop in O_2_ saturations to less than 88% or of > 5% of baseline
- A heart rate >140 bpm or a change from baseline of 20% in either direction
- An acute cardiac dysrhythmia
- Two or more signs of respiratory distress, including tachypnoea (RR >20 % from baseline), heart rate change (HR >20 % from baseline), new use of accessory muscles, abdominal paradox, new diaphoresis, or dyspnoea.

Previous MV settings were restarted if these failure criteria were met. Where patients passed SBTs, documents recommended extubation or removal of support (where tracheostomized).

# Sedation

Rates of sedative, analgesic and anxiolytic intra-venous infusions were routinely recorded every hour by clinical staff. Total daily doses (including additional bolus doses) were calculated, copied to the case report form (CRF) and subsequently entered on to the database. All sedative, analgesic and hypnotic intra-venous infusions were recorded along with number of bolus doses delivered per day. Bolus doses of sedation, analgesia or anxiolytic medications are commonly administered in response to anxiety. The frequency of these bolus doses was recorded as a surrogate marker for anxiety, in common with other research studies. Oral medications were not included. Using information from clinical reference texts [14] and published manuscripts [15, 16], opiates were converted to fentanyl equivalents using the following conversion factors:

1mg fentanyl = 0.5mg Remifentanil = 100mg morphine = 50 mg diamorphine

The Richmond Agitation and Sedation Scale (RASS) is a validated tool used for measuring depth of sedation to guide sedation management [17]. Patients are assessed and receive a score between -5 and 4 according to an ordinal, categorical scale. The score has been validated in a wide variety of patient groups [18]. Recent research has compared titration of sedation to a target RASS score against daily sedation holds, finding that titration to RASS scored is an equally effective method of sedation limitation [19]. The titration of sedative infusions to RASS scores is routine practice at the study site with scores routinely recorded by clinical staff every hour. For patients receiving MV with no indication for deep sedation, the target levels of RASS are -2 to 0. Clinical staff recorded results in the medical records; these data were copied to the CRF and subsequently entered on to the database by the investigator.

# Education and optimisation of compliance

A site initiation visit was performed prior to the commencement of the study to discuss the protocol, study procedures, the general schedule of research activities, including safety reporting procedures and good clinical practice (GCP) requirements. We provided study information packs at every bedside, identified study participation and group allocation on ventilators, and provided daily reminders of the study protocol and group allocation by research nurses. Group education was provided throughout the study during scheduled staff training events and individual sessions were provided by study investigators as needed.

# Monitoring and data recording

Research personnel recorded study data daily (Monday to Friday) using a pre-designed CRF. Data were collected until ICU discharge, death or D28, with additional follow-up for 90-day ventilator-free days (VFDs) and mortality. To determine our primary feasibility outcome, research personnel recorded time on and off assigned modes, and where available, reasons for mode cross-over. Data was transcribed from the clinical documentation at the bedside or from direct measurement and observation to the CRF, daily by study team members. These data were then entered on to a Microsoft Excel database by the investigator. Additionally, data from each ventilator were downloaded using USB drives (Servo u®™) or dedicated ventilation record cards (Servo-i®™) to confirm accuracy of documented data.

Trend (minute averaged) data, event log data and image (screen shot) data were regularly downloaded to memory cards from the ventilators. Trend data included pressure, flow, Edi, respiratory rate, delivered FiO_2_, and volume data calculated from flow. Event log data included all alarms, functions, settings and configuration. Servo-i®™ ventilators store 24 hours of data and Servo-u®™ ventilators store 72-hours of data; research staff performed the data download daily and clinical staff were requested to perform the download out-of-hours. The data were downloaded to a secure computer and copied to an Excel database for later analysis.

# Outcomes

Our primary feasibility outcome was the proportion of patients with ≥ 65% total time in the assigned MV mode (NAVA or PSV) during hours where an assisted spontaneous MV mode was used from randomization to 28 days. When designing the study, there were few data available to guide the selection of adherence level. The selection of ≥ 65% total time in the assigned MV mode was based on published compliance/adherence rates in critical care studies [20]. Secondary feasibility outcomes were (1) proportion of time spent in assigned mode; (2) proportion of patients with a protocol deviation defined as crossing over mode; (3) reasons for cross-over; (4) recruitment rate, defined as number of patients recruited per study month; and (5) protocol acceptability defined as (1) participant consent rate (those consented as a proportion of all approached) and (2) physician refusal rate (physician refusals as a proportion of those approached). To calculate the proportion of patients meeting our *a priori* compliance criteria and the proportion of time adherent to assigned mode, we discounted any time spent in CMV and Intermittent Mandatory Ventilation (IMV) modes as these were considered not to be weaning modes. We permitted CPAP use in both groups, excluding these hours from the analysis; no other exclusively spontaneously triggered MV modes were available in the participating units. We discounted crossed over hours that were within 4 hours of randomization (set-up time) and hours with PSV of ≤ 5cm H_2_O used for spontaneous breathing trials in NAVA arm patients (allowed within the protocol). We categorized reasons for assigned mode cross-over into the following categories:

1. Clinical team preference - due to lack of equipoise or due to perceived performance issues in given clinical situations
2. Edi signal problems - any Edi signal that was incompatible with safe and effective MV, either caused by clinical issues (low RASS) or technical issues (high signal interference)
3. Lack of trial awareness - when clinicians were unaware of the trial protocol
4. Clinical inexperience - non-use of NAVA due to stated inexperience with NAVA
5. NAVA catheter insertion problems; any issues that prevented the insertion of a NAVA catheter

Secondary exploratory outcomes included:

1. VFDs from randomization to D28 and D90. VFDs were the number of days participants were breathing without assistance (unassisted ventilation) during the 28-day study period, which began at the time of randomization [21, 22]. Patients who were receiving assisted ventilation at day 27 or who died during the study period were assigned zero VFDs. Unassisted breathing began at extubation to supplemental oxygen or room air; or CPAP ≤5 cm H_2_O without PS via a face-mask, helmet or tracheostomy; or removal of ventilatory support via tracheostomies. Patients receiving non-invasive PS were defined as receiving assisted ventilation. The period of unassisted ventilation needed to last for at least 48 consecutive hours. If a patient returned to assisted ventilation and subsequently achieved unassisted ventilation to D28, VFDs were counted from the end of the last period of assisted ventilation to D28. Periods of assisted ventilation lasting less than 24 hours and for a surgical procedure were not counted against the VFD calculation. If a patient was receiving assisted ventilation at day 27 or died prior to D28, they were assigned zero VFDs. Patients transferred to another hospital or other health care facility were followed to D28 to assess this endpoint.
2. Total duration of MV from randomization including, non-invasive ventilation. The successful end of ventilatory support is defined as no return to ventilatory support or death within 48 hours, and the end of ventilatory support is defined as the date and time of the final hour of ventilatory support. Non-invasive ventilation was defined as either non-invasive positive pressure ventilation (NIPPV) or Continuous Positive Airways Pressure (CPAP) > 5cm H_2_O (Table E3)
3. Time to successful extubation. Extubation from endo-tracheal intubation that is followed by ≥48 hours of spontaneous breathing. The use of NIV is counted as MV.
4. Mortality (ICU, hospital discharge, 28 and 90 days). Mortality was calculated at ICU discharge, hospital discharge, at 28-days and at 90-days from randomisation and was defined as deaths from any cause within these timeframes.
5. Length of ICU and hospital stay. Duration of ICU stay was calculated as the sum of the duration from the date and time of randomization to the date and time of discharge from the ICU or death, including all critical care unit stays where admission was within the study period. The duration of hospital stay was calculated as the duration in days from the date of randomization to the date of discharge from acute hospital or death.
6. Mean daily sedation dose per infusion day from randomization to study end. The median and interquartile range of these values is presented
7. Mean daily bolus doses administered per infusion day. The median and interquartile range of these values is presented
8. Mean RASS [17] (recorded hourly) during invasive ventilatory support. The RASS scores were recorded hourly for each patient by clinical staff. Scores recorded during IMV between randomisation and the end of IMV were totalled and divided between the number of in hours, providing a mean score for each patient. The process was then repeated using only hours where participants were in the assigned mode and only hours where participants were receiving mandatory ventilation. The median and interquartile range of these values is presented. Local unit policy and study guidelines advised clinical staff to target a RASS of between -2 and 0 during invasive ventilation, unless there was rationale for deep sedation

Safety outcomes included:

1. Ventilator associated pneumonia (developed 48 hours after randomization) determined using the Hospitals in Europe Link for Infection Control through Surveillance (HELICS) definitions [23]
2. Pneumothoraxes due to barotrauma while invasively ventilated within the study period
3. Incidence of unplanned or self extubation

Some prespecified secondary outcomes are not reported in this manuscript. For transparency, these are discussed in the table below.

| **Outcome** | **Reason planned** | **Reason not reported** |
| --- | --- | --- |
| Incidence of delirium | Due to an association with sedation load, and the potential impact of NAVA on sedation load. CAM-ICU was collected during the course of usual care at the study site. | CAM-ICU assessments were introduced to the clinical guidelines at the study site in 2012. Initially, assessments by clinical staff were inconsistent, leading to issues with data quality and missing data. Resource was not available for research staff to perform the assessments |
| Change in sequential organ failure assessment (SOFA) scores | To provide an indication of changing clinical condition over time | Baseline data is presented. Analysis is currently ongoing, but beyond timescale of reporting for the current study |
| Patient-ventilator asynchrony | Widely believed to be a mechanism underlying clinical benefit from NAVA | Catheters were not inserted in all PSV participants. |
| Electrical activity of the diaphragm (Edi) | To investigate the change in Edi over the course of prolonged ventilation | Catheters were not inserted in all PSV participants. |
| Health related quality of life at 90-days post ICU discharge (SF-36 survey) | To investigate potential long-term impact of NAVA | 31 (39.7%) surveys were successfully conducted, 37 (47.4%) were incomplete due to either death or lack of capacity, and 10 (12.8%) were lost to follow-up. Analysis is currently ongoing, but beyond timescale of reporting for the current study |

# References

1. Boles JMB, J.; Connors, A.; Herridge, M.; Marsh, B.; Melot, C.; Pearl, R.; Silverman, H.; Stanchina, M.; Vieillard-Baron, A.; Welte, T.: **Weaning from mechanical ventilation**. *Eur Respir J* 2007, **29**(5):1033-1056.

2. The Ards Definition Task Force: **Acute respiratory distress syndrome: The berlin definition**. *JAMA* 2012, **307**(23):2526-2533.

3. King's College London: **King's Clinical Trials Unit Online Randomisation Service v1.2.** [**http://www.ctu.co.uk/**](http://www.ctu.co.uk/)**.** In*.* London; 2014.

4. Thabane LM, J.; Chu, R.; Cheng, J.; Ismaila, A.; Rios, L. P.; Robson, R.; Thabane, M.; Giangregorio, L.; Goldsmith, C. H.: **A tutorial on pilot studies: the what, why and how**. *BMC Med Res Methodol* 2010, **10**:1.

5. Thabane L, Ma J, Chu R, Cheng J, Ismaila A, Rios LP, Robson R, Thabane M, Giangregorio L, Goldsmith CH: **A tutorial on pilot studies: the what, why and how**. *BMC Med Res Methodol* 2010, **10**:1.

6. Hodges JLL, E. L.: **Estimates of Location Based on Rank-Tests**. *Ann Math Stat* 1963, **34**(2):598-&.

7. Schulz KFA, D. G.; Moher, D.; Consort Group: **CONSORT 2010 statement: updated guidelines for reporting parallel group randomized trials**. *Ann Intern Med* 2010, **152**(11):726-732.

8. Gardner MJA, D G: **Confidence intervals rather than P values: estimation rather than hypothesis testing**. *British Medical Journal (Clinical research ed)* 1986, **292**(6522):746-750.

9. Cole FL: **Content analysis: process and application**. *Clinical nurse specialist CNS* 1988, **2**(1):53-57.

10. Brower RG, Matthay MA, Morris A, Schoenfeld D, Thompson BT, Wheeler A: **Ventilation with lower tidal volumes as compared with traditional tidal volumes for acute lung injury and the acute respiratory distress syndrome**. *N Engl J Med* 2000, **342**(18):1301-1308.

11. Bonmarchand G, Chevron V, Chopin C, Jusserand D, Girault C, Moritz F, Leroy J, Pasquis P: **Increased initial flow rate reduces inspiratory work of breathing during pressure support ventilation in patients with exacerbation of chronic obstructive pulmonary disease**. *Intensive Care Med* 1996, **22**(11):1147-1154.

12. Girard TD, Kress JP, Fuchs BD, Thomason JWW, Schweickert WD, Pun BT, Taichman DB, Dunn JG, Pohlman AS, Kinniry PA *et al*: **Efficacy and safety of a paired sedation and ventilator weaning protocol for mechanically ventilated patients in intensive care (Awakening and Breathing Controlled trial): a randomised controlled trial**. *The Lancet* 2008, **371**(9607):126-134.

13. Mehta S, Burry L, Cook D, Fergusson D, Steinberg M, Granton J, Herridge M, Ferguson N, Devlin J, Tanios M *et al*: **Daily sedation interruption in mechanically ventilated critically ill patients cared for with a sedation protocol: a randomized controlled trial**. *JAMA* 2012, **308**(19):1985-1992.

14. **British National Formulary**. London: PHARMACEUTICAL PRESS; 2018.

15. Patanwala AE, Duby J, Waters D, Erstad BL: **Opioid conversions in acute care**. *The Annals of pharmacotherapy* 2007, **41**(2):255-266.

16. Glass PS, Iselin-Chaves IA, Goodman D, Delong E, Hermann DJ: **Determination of the potency of remifentanil compared with alfentanil using ventilatory depression as the measure of opioid effect**. *Anesthesiology* 1999, **90**(6):1556-1563.

17. Sessler CN, Gosnell MS, Grap MJ, Brophy GM, O'Neal PV, Keane KA, Tesoro EP, Elswick RK: **The Richmond Agitation-Sedation Scale: validity and reliability in adult intensive care unit patients**. *Am J Respir Crit Care Med* 2002, **166**(10):1338-1344.

18. Ely E, Truman B, Shintani A, et al.: **Monitoring sedation status over time in icu patients: Reliability and validity of the richmond agitation-sedation scale (rass)**. *JAMA* 2003, **289**(22):2983-2991.

19. Mehta SB, L.; Cook, D.; Fergusson, D.; Steinberg, M.; Granton, J.; Herridge, M.; Ferguson, N.; Devlin, J.; Tanios, M.; Dodek, P.; Fowler, R.; Burns, K.; Jacka, M.; Olafson, K.; Skrobik, Y.; Hebert, P.; Sabri, E.; Meade, M.; Sleap Investigators; Canadian Critical Care Trials, Group: **Daily sedation interruption in mechanically ventilated critically ill patients cared for with a sedation protocol: a randomized controlled trial**. *JAMA* 2012, **308**(19):1985-1992.

20. Dodd SW, I. R.; Williamson, P.: **Nonadherence to treatment protocol in published randomised controlled trials: a review**. *Trials* 2012, **13**:84.

21. Schoenfeld DA, Bernard GR: **Statistical evaluation of ventilator-free days as an efficacy measure in clinical trials of treatments for acute respiratory distress syndrome**. *Crit Care Med* 2002, **30**(8):1772-1777.

22. McAuley DF, Laffey JG, O'Kane CM, Perkins GD, Mullan B, Trinder TJ, Johnston P, Hopkins PA, Johnston AJ, McDowell C *et al*: **Simvastatin in the Acute Respiratory Distress Syndrome**. *New England Journal of Medicine* 2014, **371**(18):1695-1703.

23. Wilson JR, I.; Suetens, C.: **Hospitals in Europe Link for Infection Control through Surveillance (HELICS). Inter-country comparison of rates of surgical site infection--opportunities and limitations**. *The Journal of hospital infection* 2007, **65 Suppl 2**:165-170.
