# Supplementary material for: Neurally adjusted ventilatory assist versus pressure support ventilation: a randomized controlled feasibility trial performed in patients at risk of prolonged mechanical ventilation
Source: Crit Care. 2020 May 14;24:220. doi: 10.1186/s13054-020-02923-5 (PMC7224141; doi:10.1186/s13054-020-02923-5)
Supplement: Supplementary file 2 — Additional file 2. Supplemental data tables. [file 13054_2020_2923_MOESM2_ESM.docx]

**Additional file 2: Supplemental data tables**

**Article title**

Neurally Adjusted Ventilatory Assist versus Pressure Support Ventilation: A Randomized Controlled Feasibility Trial

**Authors**

Daniel J. Hadfield ^1,2^, Louise Rose ^3,4^, Fiona Reid ^5^, Victoria Cornelius ^6^, Nicholas Hart ^2,7^, Clare Finney ^1^, Bethany Penhaligon ^1^, Jasmine Molai ^1^, Clair Harris ^1^, Sian Saha ^1^_,_ Harriet Noble ^1^, Emma Clarey ^1^, Leah Thompson ^1^, John Smith ^1^, Lucy Johnson ^1^, Phillip A. Hopkins ^1^ and Gerrard F. Rafferty ^2^

^1^ Critical Care, King's College Hospital, London, United Kingdom

^2^ Centre for Human and Applied Physiological Sciences, King's College London, United Kingdom

^3^ Florence Nightingale Faculty of Nursing, Midwifery & Palliative Care, King’s College London, United Kingdom

^4^ Sunnybrook Health Sciences Centre and Sunnybrook Research Institute, Toronto, Canada

^5^ School of Population Health and Environmental Sciences, King's College London, London, United Kingdom

^6^ Faculty of Medicine, School of Public Health, Imperial College

^7^ Lane Fox Unit, Guy's and St Thomas' NHS Foundation Trust, London, England

**Corresponding Author:**

Daniel Hadfield. Email: daniel.hadfield@nhs.net.

**Contents**

[Table 1. Trial exclusions 3](#_Toc29205835)

[Table 2. Duration of MV, ICU and hospital stay results for survivors and non-survivors 4](#_Toc29205836)

[Table 3. Glossary of ventilation terms 5](#_Toc29205837)

[Table 4. Protocol amendments 6](#_Toc29205838)

[Table 5. Additional ventilation data 7](#_Toc29205839)

[Table 6. Participants who did not ventilate in the assigned mode 8](#_Toc29205840)

[References 9](#_Toc29205841)

# Table 1. Trial exclusions

| Reason for exclusion | Number |
| --- | --- |
| Suspected or proven hypoxic brain injury | 108 (13.9%) |
| Extubation planned within 48 hours | 104 (13.4%) |
| Patient likely to die or have treatment withdrawal within 48 hours | 85 (11%) |
| Greater than 96 hours from intubation (unavailability of researchers) | 70 (9%) |
| In PSV mode for >24 hours | 52 (6.7%) |
| Contraindication to passing NG tube | 41 (5.3%) |
| Enrolled in another clinical trial in the last 30 days | 38 (4.9%) |
| Consultee declined assent | 31 (4%) |
| Physician refusal | 15 (1.9%) |
| High spinal injury / severe TBI | 14 (1.8%) |
| Hepatic encephalopathy | 12 (1.5%) |
| Domiciliary mechanical ventilation * | 8 (1%) |
| Does not understand verbal or written information | 2 (0.3%) |
| Pregnant | 1 (0.1%) |
| In PSV mode for >24 hours | 34 (4.4%) |
| Unknown | 29 (3.7%) |
| Existing lack of capacity to consent | 20 (2.6%) |
| Researcher unavailable | 16 (2.1%) |
| Vulnerable adult † | 16 (2.1%) |
| Psychiatric issue | 6 (0.8%) |
| Neurological cause of ventilator dependence ‡ | 5 (0.6%) |
| Not eligible for NHS treatment | 4 (0.5%) |
| Consent provided outside permitted window | 3 (0.4%) |
| For transfer | 3 (0.4%) |
| Active cardiac pacing | 1 (0.1%) |
| NG = naso-gastric; PSV = Pressure Support Ventilation; TBI = traumatic brain injury  * excluding ventilation for disordered sleep  † safeguarding issues or family/social complexity preventing informed consent  ‡ such as Guillain-Barré syndrome or Myasthenia Gravis  § Patients may experience >1 reason for cross-over, therefore the sum of percentages is not 100% | |

# Table 2. Duration of MV, ICU and hospital stay results for survivors and non-survivors

| Variable | NAVA (n=39) | PSV (n=38) | Effect estimates | P value |
| --- | --- | --- | --- | --- |
| ***Continuous variables (median, IQR, n)*** | | | ***MD (95% CI)*** |  |
| Duration of MV, d* | 4.9 (2.8–15.7) | 9.8 (3.6–106.3) | 3 (-0.4–8.6) | 0.094 |
| Duration of MV (survivors), d* | 8.6 (5.6–18.4), 30 | 10.7 (4.4–25.5), 23 | 1 (-2.7–6.9) | 0.554 |
| ICU stay, d | 9.1 (6–21.9) | 14.8 (7–33.1) | 3.3 (-1.1–8.8) | 0.158 |
| ICU stay (survivors), d | 9.2 (7–24), 31 | 14.2 (7–30.7), 23 | 1.6 (-3.7–8.2) | 0.588 |
| Hospital stay, d | 19.9 (11.9–42.8) | 26.6 (11.3–61.1) | 4 (-5–14.9) | 0.419 |
| Hospital stay (survivors), d | 25 (15.6–52.8), 30 | 29.6 (16.8–63.9), 19 | 4.1 (-7.3–18.9) | 0.522 |
| All durations measured from randomization. NAVA = Neurally Adjusted Ventilatory Assist; PSV = Pressure Support Ventilation; IQR = interquartile range; MD = median difference, calculated using the Hodges Lehmann estimating method [1]; CI = confidence interval  * excludes patient 76 who had prior home dependence on bi-level ventilation. | | | | |

# Table 3. Glossary of ventilation terms

| **Term** | **Definition** |
| --- | --- |
| Mechanical ventilation (MV) | Any invasively or non-invasively delivered ventilation support ≥5 cm H_2_0 of PEEP. CPAP support not exceeding 5cm H2O via mask or tracheostomy not be counted as MV |
| Continuous mandatory ventilation (CMV) | Any ventilation mode where all inspirations are mandatory |
| Intermittent mandatory ventilation (IMV) | Any mode where spontaneous breaths are allowed above a mandatory set breathing rate |
| Continuous, spontaneously triggered ventilation mode (CSV) | Modes where there is no mandatory set breathing rate, and additional support above PEEP is triggered by patient inspiration |
| Non-invasive positive pressure ventilation (NIPPV) | Ventilatory support delivered via mask or hood without the need for tracheal intubation |
| Continuous positive pressure ventilation (CPAP) | Continuous, single level positive pressure. Can be delivered invasively or non-invasively |
| Non-invasive ventilation (NIV) | NIPPV or CPAP > 5cm H2O delivered via mask or hood without the need for tracheal intubation |
| PEEP = positive end expiratory pressure | |
|  |  |

# Table 4. Protocol amendments

| **Type / date** | **Detail** |
| --- | --- |
| HRA amendment  17/10/2016 CT registry changed 7/12/2016 | **Change:** New sample size rationale and a reduction in the total recruitment target from 92 to 76  **Rationale:** Peer review from the NIHR and others suggested that the sample size calculation and supporting rationale required updating in light of literature published after the commencement of the study [2, 3] |
| HRA amendment  17/10/2016 CT registry changed 7/12/2016 | **Change:** Feasibility and secondary outcomes clarified  **Rationale:** When entered on the public trials registry (www.clinicaltrials.com), ventilator-free-days was incorrectly added in the primary outcome field. This was an error. The trial was originally designed to assess feasibility; the aims are consistently presented in the study in the protocol and in the title and text of the public trials registry |
| HRA amendment  04/12/2014  CT registry changed 7/12/2016 | **Change:** Alteration to inclusion criteria to remove patients with interstitial lung disease and include patients with Acute Respiratory Distress Syndrome (ARDS)  **Rationale:** The study aimed to recruit patients at risk of prolonged mechanical ventilation in the ICU. Interstitial lung disease was removed due to the overall poor prognosis of ventilated ILD patients. ARDS was included following research demonstrating an association with prolonged ventilator weaning [4] and a new international consensus on the definition of ARDS [5] published after the trial registration. |
| HRA amendment  04/12/2014  No change to CT registry | **Change:** Removal of the requirement for PSV arm participants to receive a NAVA catheter.  **Rationale:** The study protocol initially advised the placement of NAVA catheters in all patients. It became apparent, that clinicians were reluctant to remove existing NG feeding catheters and insert NAVA catheters in the sickest PSV arm participants. The collection of Edi data was secondary to the main trial objectives, and it was hoped that removal of this requirement would prevent selection bias. |
| HRA = United Kingdom Health Research Authority; NIHR = National Institute for Health Research (UK);  CT = clinicaltrials.com | |

# Table 5. Additional ventilation data

| Variable | NAVA group* | PSV group* |
| --- | --- | --- |
| *Values from combined NAVA and PSV mode hours (including cross-over)* | | |
| Tidal volume, ml | 452 (385 to 511), 32 | 516 (433 to 588), 30 |
| Tidal volume, ml/kg | 6.1 (4.8 to 7.5), 32 | 6.6 (5.6 to 8.4), 30 |
| Minute ventilation | 10.3 (8.9 to 11.2), 32 | 9.7 (8.3 to 10.9), 30 |
| Respiratory rate | 22.3 (17 to 25.8), 32 | 18.6 (15 to 21.5), 30 |
| FiO_2_ | 34.4 (29.2 to 38.2), 32 | 30.6 (26.4 to 35.6), 30 |
| PEEP | 8.1 (6.8 to 9.7), 32 | 7.6 (6.3 to 9.6), 30 |
| Peak inspiratory pressure | 18.9 (17.3 to 21.2), 32 | 19.2 (16.8 to 21.7), 30 |
| Mean inspiratory pressure | 10.8 (9.5 to 11.5), 32 | 10.8 (9.2 to 12.0), 30 |
| *Values in the assigned mode (NAVA versus PSV - per protocol)* | | |
| Tidal volume, ml | 435 (357 to 479), 30 | 514 (433 to 578), 30 |
| Tidal volume, ml/kg | 5.8 (4.6 to 6.5), 30 | 6.6 (5.7 to 8.3), 30 |
| Minute ventilation | 10.1 (8.8 to 11.4), 30 | 9.7 (8.2 to 10.7), 30 |
| Respiratory rate | 22.9 (21.1 to 26.4), 30 | 18.7 (16.1 to 21.5), 30 |
| FiO_2_ | 32.6 (28.7 to 38), 30 | 30.6 (26.4 to 35.6), 30 |
| PEEP | 8.4 (7.3 to 9.6), 30 | 7.6 (6.3 to 9.6), 30 |
| Peak inspiratory pressure | 18.8 (16.7 to 21.4), 30 | 19.2 (16.6 to 21.6), 30 |
| MV > 21 days (survivors), n (%) | 7/32 (12.5%) | 11/28 (39.3%) |
| MV on D28 (survivors), n (%) | 4/31 (5.1%) | 9/27 (18.4%) |
| MV on D90 (survivors), n (%) | 1/30 (3.2%) | 1/21 (3.7%) |
| Death on invasive MV, n (%) | 8/39 (20.5) | 7/38 (18.4) |
| *Data not collected in four participants in the NAVA arm and six participants in the PSV arm due to failed electronic data capture. All durations measured from randomization. NAVA = Neurally Adjusted Ventilatory Assist; PSV = Pressure Support Ventilation; PEEP = positive end expiratory pressure; IQR = interquartile range; MV = mechanical ventilation defined as any ventilation support via an endotracheal tube, or tracheal or non-invasive ventilation > 5 cm H_2_0 of CPAP | | |

# Table 6. Participants who did not ventilate in the assigned mode

| Participant | Randomized group | Hours of crossed over mode | Reason |
| --- | --- | --- | --- |
| 01043 | NAVA | 0 | Died on CMV |
| 01047 | NAVA | 0 | Extubated from CMV. Died |
| 01069 | NAVA | 0 | Died on CMV |
| 01029 | NAVA | 7 | Due to trial awareness and lack of experience out of hours, patient was weaned in PSV and extubated after 7 hours |
| 01036 | NAVA | 12 | Edi signal problems. Unable to acquire a useable Edi signal, free from electrical interference |
| 01040 | NAVA | 12 | Edi signal problems. Edi low and unable to acquire a useable signal, free from electrical interference |
| 01004 | PSV | 0 | Died on CMV |
| 01039 | PSV | 0 | Died on CMV |
| NAVA: Neurally Adjusted Ventilatory Assist; PSV = Pressure Support Ventilation; Edi = diaphragmatic electrical activity; CMV = continuous mandatory ventilation | | | |

# References

1. Hodges JLL, E. L.: **Estimates of Location Based on Rank-Tests**. *Ann Math Stat* 1963, **34**(2):598-&.

2. Eldridge SML, G. A.; Campbell, M. J.; Thabane, L.; Hopewell, S.; Coleman, C. L.; Bond, C. M.: **Defining Feasibility and Pilot Studies in Preparation for Randomised Controlled Trials: Development of a Conceptual Framework**. *PLoS One* 2016, **11**(3):e0150205.

3. Eldridge SM, Chan CL, Campbell MJ, Bond CM, Hopewell S, Thabane L, Lancaster GA, Grp PC: **CONSORT 2010 statement: extension to randomised pilot and feasibility trials**. *Bmj-Brit Med J* 2016, **355**.

4. Topfer L, Menk M, Weber-Carstens S, Spies C, Wernecke KD, Uhrig A, Lojewski C, Jorres A, Deja M: **Influenza A (H1N1) vs non-H1N1 ARDS: Analysis of clinical course**. *J Crit Care* 2014, **29**(3):340-346.

5. The Ards Definition Task Force: **Acute respiratory distress syndrome: The berlin definition**. *JAMA* 2012, **307**(23):2526-2533.
